# Predicting oral malodour based on the microbiota in saliva samples using a deep learning approach

**DOI:** 10.1186/s12903-018-0591-6

**Published:** 2018-07-31

**Authors:** Yoshio Nakano, Nao Suzuki, Fumiyuki Kuwata

**Affiliations:** 1 Department of Chemistry, Nihon University School of Dentistry, Kanda-Surugadai, Chiyoda-ku, Tokyo, 101-8310 Japan; 2 Department of Preventive and Public Health Dentistry, Fukuoka Dental College, Tamura, Sawara-ku, Fukuoka, 814-0193 Japan

**Keywords:** Oral malodour, Deep learning, Oral micorobiota

## Abstract

**Background:**

Oral malodour is mainly caused by volatile sulphur compounds produced by bacteria and bacterial interactions. It is difficult to predict the presence or absence of oral malodour based on the abundances of specific species and their combinations. This paper presents an effective way of deep learning approach to predicting the oral malodour from salivary microbiota.

**Methods:**

The 16S rRNA genes from saliva samples of 90 subjects (45 had no or weak oral malodour, and 45 had marked oral malodour) were amplified, and gene sequence analysis was carried out. Deep learning classified oral malodour and healthy breath based on the resultant abundances of operational taxonomic units (OTUs)

**Results:**

A discrimination classifier model was constructed by profiling OTUs and calculating their relative abundance in saliva samples from 90 subjects. Our deep learning model achieved a predictive accuracy of 97%, compared to the 79% obtained with a support vector machine.

**Conclusion:**

This approach is expected to be useful in screening the saliva for prediction of oral malodour before visits to specialist clinics.

**Electronic supplementary material:**

The online version of this article (10.1186/s12903-018-0591-6) contains supplementary material, which is available to authorized users.

## Background

Oral malodourous compounds are reportedly produced by periodontitis-associated bacteria in the oral cavity, such as those belonging to the genera *Porphyromonas* and *Prevotella*, which produce volatile sulphur compounds (VSCs) [1–4]. *Fusobacterium nucleatum* and *Treponema denticola* also produce VSCs, but additionally produce butyric acid and other volatile organic compounds that cause oral malodour [3, 5–7]. Nevertheless, because there are over 700 known bacterial species in the human oral cavity [8, 9], and because several species produce VSCs to varying degrees, the presence of VSCs in the breath cannot be predicted by the presence of specific species. Concentrations of oral malodourous compounds produced by oral bacteria vary according to the type and abundance of species. Interactions between bacterial species may play important roles in the production of VSCs.

Analysis of the oral microbiota reveals several signals from various bacterial species present in various numbers, but we cannot directly or indirectly distinguish bacterial species producing oral malodourous compounds from non-producing bacteria; the bacterial cells form complicated networks in the oral cavity. Machine learning is suitable for prediction from such complicated data, and we previously reported some success in predicting oral malodour using support vector machines (SVMs) [10].

Machine learning algorithms use training data to uncover underlying patterns, build models, and make predictions based on the best fit models. Indeed, some well-known algorithms, such as SVMs, random forests, Bayesian networks, and Gaussian networks, have been applied in genomics, proteomics, systems biology, and numerous other domains [11]. We previously reported prediction of oral malodour from oral microbiota in saliva by using an SVM based on peak areas of terminal restriction fragment length polymorphisms (T-RFLPs) of the 16S rRNA gene as data for supervised machine-learning methods [10]. Using this training data, the SVM achieved a high classification accuracy of 82%, with a sensitivity of 51% and specificity of 95%. Currently, T-RFLP does not provide economic advantages over 16S RNA sequence analysis. In this study, we devised a more precise classification system using a deep-learning approach based on 16S rRNA sequences with a higher resolution than that of T-RFLP analysis, and compared it with SVM-based prediction.

## Methods

### Study population

The study population consisted of 90 patients (37 men and 53 women, mean age of 50.0 ± 14.7 years) who had visited the Oral Malodour Clinic of Fukuoka Dental College Medical and Dental Hospital between August 2011 and October 2016 with a complaint of halitosis. They had not consumed antibiotics within 3 months and had no otorhinolaryngological illness or metabolic disease. Of the 90 patients, 45 had no or weak oral malodour and 45 had marked oral malodour. All participating subjects understood the nature of the research project and provided written, informed consent. Permission for this study was obtained from the Ethics Committee for Clinical Research of Fukuoka Dental College and Fukuoka College of Health Sciences (approval numbers 89, 233, and 249). All study methods were carried out in accordance with the approved guidelines.

### Malodour assessment

The severity of oral malodorour was determined in each patient using an organoleptic test (OLT) and gas chromatography. Malodour assessment and clinical examination, including tests of salivary flow and mucosal moisture level, were performed at least 5 h after eating, drinking, chewing, smoking, and brushing or rinsing the mouth. The OLT scores were estimated by two of three evaluators using a scale of 0 to 5 [12], and the mean of the scores given by the evaluators was used. The presence of OLT scores ≥ 2 among the three evaluators always exceeded 75% (= 0.50). Gas chromatography (model GC2014; Shimazu Works, Kyoto, Japan) was used to measure the concentrations of hydrogen sulphide (*H*_2_S), methyl mercaptan (C*H*_3_SH), and dimethyl sulphide (C*H*_3_*SCH*_3_) in the breath. The value for total VSCs was defined as the sum of the *H*_2_S, *CH*_3_SH, and *CH*_3_*SCH*_3_ concentrations. The threshold for marked oral malodour was defined as an OLT score of ≥ 3 and total VSC concentration of ≥ 0.3 ppm. The threshold for no or weak oral malodour was defined as an OLT score of < 3 and total VSC concentration of < 0.3 ppm.

### Sample collection and pyrosequencing analysis

Saliva samples were collected from subjects using chewing gum. Subjects were asked to spit into a vessel throughout the a 5 min collection period. Samples (0.5 ml) were collected and were transferred to sterile plastic tubes. Bacteria were harvested by centrifugation (20,400 × g, 15 min at 4°C), and the resulting pellets were resuspended in 150 *μ*l of buffer containing 50 mM Tris-HCl, 1 mM EDTA, and 1% sodium dodecyl sulfate (SDS; pH 7.6). The suspension was added to plastic tubes containing 0.3 g zirconia-silica beads (bead size, 0.1 mm; Biospec Products, Bartlesville, OK, USA) and one tungsten-carbide bead (bead size, 3 mm; Qiagen, Hilden, Germany). The samples were heated at 90°C for 10 min and then vigorously agitated for 3 min in a cell disruptor (Disruptor Genie, Scientific Industries, Inc., Bohemia, NY, USA). After centrifugation at 6000 × g for a few seconds, 200 *μ*l of 1% SDS was added, and the samples were incubated at 70°C for 10 min. The mixtures were extracted using 400 *μ*l of phenol–chloroform–isoamyl alcohol (25:24:1), and the nucleic acids were precipitated with 100% ethanol. Following centrifugation, the DNA was washed with 70% ethanol, resuspended in 100 *μ*l of TE buffer (10 mM Tris-HCl, 1 mM EDTA, pH 7.6), and frozen until subsequent analysis. After extraction, samples were PCR-amplified under permissive conditions using primers to amplify the 508-807 region in prokaryotic 16S rDNA containing the MiSeq sequencing adapters and an 8-nucleotide barcode on the forward primer, followed by the bases matching the 16S rRNA gene. The analysis was performed using the forward primer AA TGA TAC GGC GAC CAC CGA GAT CTA CAC XXXXXXXX TCG TCG GCA GCG TCA GAT GTG TAT AAG AGA CAG and the reverse primer GTT CGT CTT CTG CCG TAT GCT CTA CAA GCA GAA GAC GGC ATA CGA GAT XXXXXXXX CAG AGC ACC CGA GCC TCT ACA CAT ATT CTC TGT C. Pyrosequencing was conducted at Hokkaido System Science Co., Ltd. (Sapporo, Japan) on an Illumina MiSeq sequencer (Illumina, San Diego, CA, USA) using a paired-end 300 bp sequence read run with the Miseq Reagent Kit v3 and MiSeq Control Software version 2.6.2.1 (Illumina).

### Data analysis and taxonomy assignment

Putative chimera sequences were removed by UCHIME v6.1.544 [13], and sequences with 80% of their nucleotides of fragment quality score 20 or lower were removed. The remaining sequences were assigned to OTUs using cd-hit with a 98% threshold of pairwise identity [14]. Each representative sequence was compared using the BLAST algorithm with 998 sequences of the oral bacterial 16S rRNA gene (Human Oral Microbiome Database [HOMD] 16S rRNA RefSeq Version 15.1) deposited in HOMD and assigned to the best BLAST hit with a 97% identity. A total of 3000 sequences were randomly extracted from each sample and used for the following analyses (Additional files 1 and 2). LDA effect size (LEfSe) [15] analysis was used to detect significant differences between the relative abundances of OTUs in samples from patients with healthy and malodourous breath.

### Machine learning

Learning and classification of the bacterial composition of each sample were accomplished using R (http://www.r-project.org) with the h2o package for deep learning and the activation type RectifierWithDropout, and the e1071 package for the SVM with the radial basis function (RBF). The radial kernel function transformed the data using the non-linear function *k*(*x*1,*x*2)=*exp*(−*γ*|*x*1−*x*2|2), where *γ* determines the RBF width, unless otherwise specified. Classification by machine learning was evaluated by leave-one-out cross-validation, i.e., one sample was classified by supervised machine learning using the other 89 samples for training. The commands used in this study are showed in Additional file 3.

## Results

### Evaluation of microbiome compositions based on 16S rRNA sequences

**Fig. 1:**
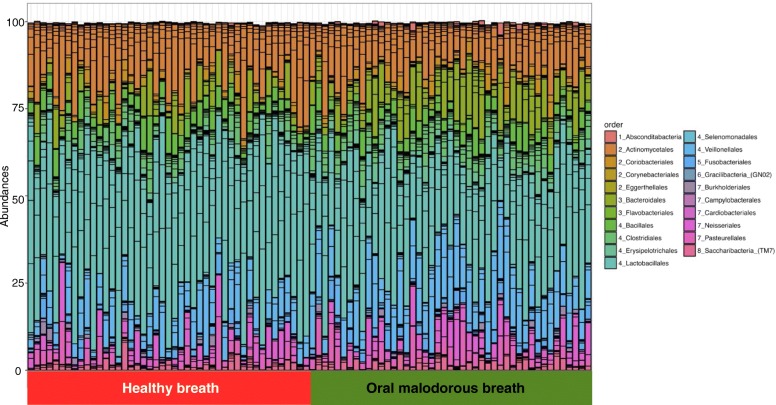
Bar plot of abundance of orders in each sample using a phyloseq package. The orders are ordered by phylum: 1. Absconditabacteria; 2. Actinobacteria; 3. Bacteroidetes; 4. Firmicutes; 5. Fusobacteria; 6. Gracilibacteria (GN02); 7. Proteobacteria; 8. Saccharibacteria (TM7)

Nucleotide sequences of 16S rRNAs were determined and their taxa were estimated by BLAST analysis using HOMD. A total of 3000 sequences from each sample were analysed and the results showed a typical bacterial composition profile (Fig. 1) when compared to the 16S rRNA sequences of known oral microbes. OTUs with ≤ 0.1% frequency, found in ≤ 4 samples, were omitted from the following calculation. In total, 108 distinct OTUs were noted, and the minimum, maximum, and mean numbers of OTUs per sample were 20, 66, and 38.9, respectively (Additional file 1).

Some OTUs belonged to the same genus, so we re-examined the composition of the genera (Additional file 2). Thirty-seven were present in the samples. Genera characteristic of healthy and malodourous mouth breath were analysed using the Mann-Whitney U test. Table 1 shows the significance of bacteria between two groups.

**Table 1:** Malodourous and healthy breath-specific genera compared with non-parametric Mann Whitney U test (*p*<0.05)

	*p*	Malodourous group (%)	Healthy group (%)
Streptococcus	3.9×10^−6^	25.6	34.9
Granulicatella	0.0012	4.50	6.67
Cryptobacterium	0.0066	0.03	0.07
Rothia	0.011	9	12.37
Prevotella	3.3×10^−7^	3.60	0.90
Veillonella	2.0×10^−5^	13.6	8.73
Peptostreptococcus	7.7×10^−5^	21.2	1.23
Peptostreptococcaceae	0.00044	0.98	0.59
Megasphaera	0.0011	0.36	0.15
Leptotrichia	0.0035	2.32	1.57
Absconditabacteria	0.0040	0.38	0.060
Porphyromonas	0.0076	5.80	3.60
Capnocytophaga	0.011	0.49	0.22
Stomatobaculum	0.014	0.29	0.19
Eikenella	0.023	0.04	0.01
Solobacterium	0.023	2.07	1.44
Parvimonas	0.032	0.65	0.42

**Fig. 2:**
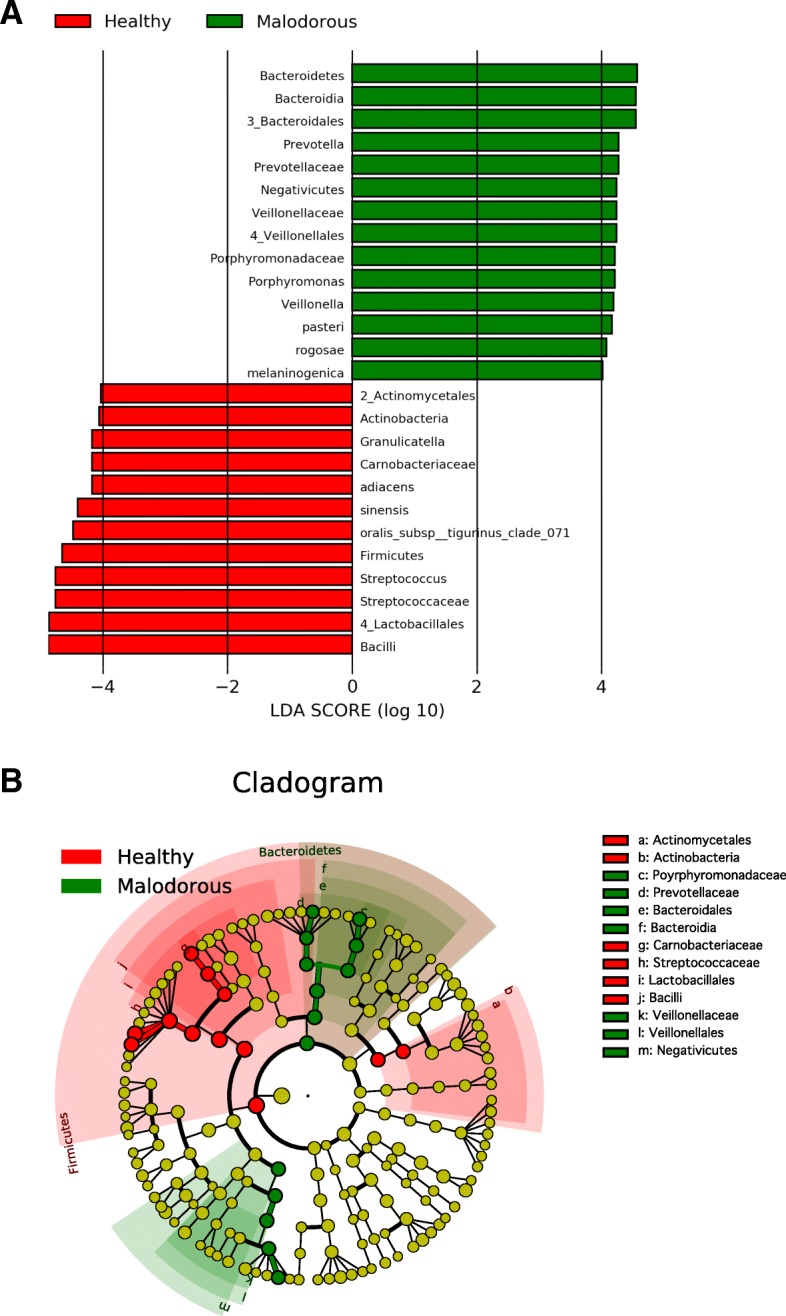
LEfSe analysis. **a** Histogram of the LDA scores computed for features differentially abundant in healthy (red) and oral malodourous (green) breath. **b** Cladogram showing different abundance values (according to LEfSe) of taxa

Linear discriminant analysis (LDA) was performed using LEfSe [15] to detect OTUs with significantly different relative abundances in oral malodourous and healthy breath. A total of 108 OTUs were found to be significantly differences between bacteria in the groups (Fig. 2). OTUs identified as most strongly associated with oral malodour were from the *Bacteroides*, *Prevotella*, and *Porphyromonas* genera.

### Classification of the presence of oral malodour by SVM and deep learning

To evaluate the classification performance of SVM and deep learning for oral malodour, proportions of OTUs associated with oral malodour in 16S rRNA analysis were used for classification by the support vector machine and deep learning (Table 2). Deep learning discriminated oral malodour from normal breath with 96.7% accuracy as compared to SVM, which discriminated between them with 78.9% accuracy.

**Table 2:** Recognition rates of oral malodour by SVM and deep learning

	Sensitivity (%)	Specificity (%)	Accuracy (%)
SVM	77.8	80.0	78.9
Deep learning	100	93.3	96.7

## Discussion

We previously reported that SVM discriminated oral malodour from normal breath based on T-RFLP analysis with 81% accuracy [10]. T-RFs are generated by digestion with restriction enzyme(s); therefore, the resolution for discrimination of bacterial species is limited by the size of recognition sites within the target fragments and lacks quantitative ability. The costs for 16S rRNA sequence analyses have been reduced by pyrosequencing and other next-generation sequencing techniques. We expected that the higher resolution would improve the recognition rate by SVM and other machine learning systems. Contrary to our expectations, the SVM classifier discriminated between the presence and absence of oral malodour using the amplified 16S rRNA sequences from the saliva samples with an accuracy of 78.9%, similar to that of T-LFs (Table 2).

The bacterial composition in the saliva samples showed a typical oral microbiota profile (Fig. 1), and statistical analysis showed some genera specific to healthy and malodourous breath (Table 1). Of the genera present at over 5% abundance, *Streptococcus*, *Granulicatella* and *Rothia* were more abundant in the healthy group, whereas *Veillonella*, *Peptostreptococcus*, and *Porphyromonas* were more abundant in the malodourous group. These genera contain oral malodour-associated species, and *Porphyromonas* spp, in particular, as periodontal bacteria, are strongly suggested to be an oral malodour-producing bacteria [4, 16, 17]. In addition, LEfSe analysis revealed many bacterial species, genera, and families with significantly different relative abundances in oral malodourousand healthy breath. A total of 74 OTUs or OTU groups were noted to be significantly differentially abundant in these groups, with an LDA threshold of 4.0 (Fig. 2). The Bacteroidales order, which includes the genera *Prevotella* and *Porphyromonas*, was strongly associated with oral malodour. The results of LEfSe analysis include a cladogram showing the significant differences at different hierarchical levels (Fig. 2b). In this study, the relative abundance of *Fusobacterium* was an averaged 0.12%, and *Treponema* was detected in almost no samples. Thus, VSC concentrations cannot be predicted based on the abundances of some known VSC-producing bacteria, and machine learning techniques are useful.

A large proportion of oral microbiota, particularly in the saliva, does not produce oral malodourous compounds, but may indicate the presence of organisms specific to oral malodour owing to the interactions among bacterial species. Thus, machine learning can be expected to classify oral microorganisms as oral malodourous or non-malodourous, although our first trial with SVM did not show high classification accuracy (Table 2). We next focused on deep learning, which has advanced significantly in solving problems that have resisted the best attempts of the artificial intelligence community for several years [18]. Application of deep learning in bioinformatics has become a focal point of research [19].

**Fig. 3:**
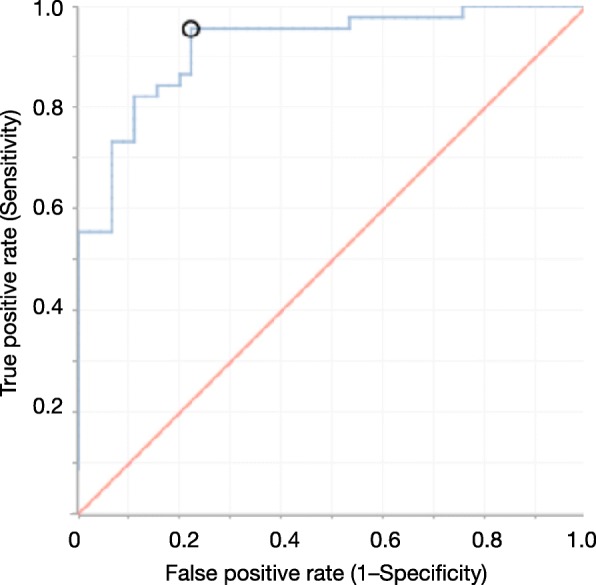
ROC curve for classification of malodourous and healthy breath using 30-fold validation with activation of Tanh

Deep learning classified oral malodour and healthy breath with 97% accuracy, improving accuracy by 15% over that obtained by SVM classification (Table 2). Notably, the sensitivity is 100%, or false negative rate is 0%. That is, anyone whose oral microbiota is classified as oral malodourous has a high risk of oral malodour. Classification with high sensitivity is desirable for screening or preliminary tests because of the lower number of false negatives, though 97% accuracy is not always expected for such biological data. ROC curve analysis (Fig. 3) suggested that this approach is effective and reliable. Patients concerned about oral malodour could send saliva samples for analysis before medical treatment. Packaging breath is impractical, whereas preparation of a saliva sample is simple, as DNA from oral bacterial cells in the saliva can be dried and transported at room temperature by using FTA cards (GE Healthcare, Little Chalfont, UK).

## Conclusions

We demonstrated deep learning-based classification of oral malodourous and healthy breath with high accuracy (97%) based on profiling of 16S rRNA sequences from microbiota in saliva samples. Classification using saliva samples is suitable for screening of oral malodour risk. In addition, treatment for oral malodour can be monitored using this classification system.

## Additional files

Additional file 1 Dataset of numbers of OTUs in samples. A total of 3000 sequences were randomly extracted from each sequence data of a sample. The second column, “Malodour”, shows malodourous (P, positive) or normal (N, negative) breath. (CSV 24 kb)

Additional file 2 Dataset of numbers of genera in samples. OTUs in Additional file 1 were combined into genera and counted again. (CSV 10 kb)

Additional file 3 The commands of h2o and e1071 in R. (TXT 1 kb)

## Abbreviations

LDA: Linear discriminant analysis
LEfSe: LDA effect size
OLT: Organoleptic test
OTU: Operational taxonomic units
RBF: Radial basis function
ROC: Receiver operating characteristic
SVM: Support vector machine
T-RFLP: Terminal restriction fragment length polymorphisms
VSC: Volatile sulphur compound
